# Effectiveness of biofeedback on blood pressure in patients with hypertension: systematic review and meta-analysis

**DOI:** 10.1038/s41371-024-00937-y

**Published:** 2024-08-14

**Authors:** Sian Jenkins, Ainslea Cross, Hanad Osman, Farah Salim, Dan Lane, Dennis Bernieh, Kamlesh Khunti, Pankaj Gupta

**Affiliations:** 1 Department of Population Health Sciences, University of Leicester, Leicester, UK; 2 Diabetes Research Centre, College of Life Sciences, University of Leicester, Leicester, UK; 3 School of Psychology and Vision Sciences, University of Leicester, Leicester, UK; 4 Department of Cardiovascular Sciences, University of Leicester, Leicester, UK; 5 Department of Metabolic Medicine & Chemical Pathology, University Hospitals of Leicester NHS Trust, Leicester, UK

**Keywords:** Hypertension, Health care

## Abstract

Hypertension is the leading modifiable risk factor for cardiovascular disease, but less than 50% have their blood pressure controlled. A possible avenue to support hypertension management is a holistic approach, using non-pharmacological interventions. Since hypertension is mediated in part by dysregulation of the autonomic nervous system (ANS), biofeedback may help improve hypertension management by targeted self-regulation and self-awareness of parameters that regulate the ANS. This systematic review aimed to assess the effectiveness of biofeedback on blood pressure in hypertensive patients. The review was pre-registered on PROSPERO and followed the PICO strategy. A total of 1782 articles were retrieved, 20 met the inclusion criteria. Sample sizes ranged from 15 to 301 participants; with a median age of 49.3 (43.3–55.0) years and 45% were female. There was a significant effect of biofeedback on systolic (−4.52, Z = 2.31, *P* = 0.02, CI [−8.35, −0.69]) and diastolic blood pressure (−5.19, Z = 3.54, *P* = 0.0004, CI [−8.07, −2.32]). Six different biofeedback modalities were used, with biofeedback delivered by psychologists, trained therapists and research assistants. There was no publication bias, heterogeneity was rated as substantial and data quality was rated to be poor. This review demonstrated that biofeedback had a significant effect on blood pressure. However, this should be viewed in the context of included studies being limited by heterogeneity and dated literature, meaning the research does not reflect the current biofeedback technology such as wearable devices. Future research should incorporate these technologies with robust methodology to fully understand the effect of biofeedback on hypertension.

## Introduction

Hypertension is the leading modifiable risk factor for cardiovascular disease, stroke and premature death [1]. Globally, 1.2 billion people have hypertension, a figure that doubled between 1990 and 2019 [1]. Worldwide hypertension control remains poor with only 21% of men and 18% of women achieving blood pressure targets [1]. This is despite the availability of cheap and effective medications. Hence it would be useful to consider non-pharmacological therapies that, in conjunction with medications, may help improve blood pressure in a more holistic manner.

It is accepted that hypertension is in part due to a derangement in the regulation of the autonomic nervous system (ANS). The sympathetic nervous system leads to increase in heart rate and blood pressure, whereas the parasympathetic nervous system relaxes the body and decreases blood pressure [2]. Hypertension is also linked to impaired baroreceptor regulation with interrelationships between baroreflex sensitivity and autonomic dysfunction [3]. There is evidence that non-pharmacological treatments such as lifestyle interventions and weight loss have a positive impact on the ANS [2]. Therefore, improved regulation in the ANS, especially an increase in parasympathetic activity, can improve blood pressure and biofeedback may help to achieve this [4].

Biofeedback improves ANS control as it promotes self-regulation, induces a ‘relaxation response’ and reduces cognitive avoidance (i.e., avoiding thoughts of undesirable situations through distraction, thought suppression or worry [5]) through increasing awareness of physiological processes [6]. Biofeedback uses instruments to measure physiological responses such as heart rate variability, sharing this with the user in real time with the aim to increase awareness and health [7]. Biofeedback is often paired with interventions that address behaviour, emotion and thoughts, which can benefit physiological processes [7]. Frank et al. [8] described biofeedback as “training not treatment” highlighting the level of motivation and practice required by the user to achieve the benefits of biofeedback. Ultimately, the goal is that these learned processes become automatic and individuals do not require device feedback to achieve the desired outcomes.

Over the years, the field of biofeedback has progressed with advances in technology. Available devices are user friendly and wearable [9–12], making biofeedback a more accessible intervention. Additionally, using a wearable device gives insight into an individual’s physiology and response to stress and daily life on a continuous basis. This is more representative than data provided by a static clinic blood pressure measurement. With the improvement of technology, accessibility to biofeedback and progressions in artificial intelligence (AI), it is important to understand the existing literature and how we can progress knowledge and implementation of biofeedback to improve health and wellbeing. This review aimed to assess the effectiveness of biofeedback in patients with hypertension. The main outcome assessed was a change in blood pressure.

## Methods

### Eligibility criteria

This review was pre-registered on PROSPERO (ID: CRD42021285875) and follows PRISMA 2020 reporting guidelines [13]. Inclusion criteria were as follows: assessment of biofeedback (all modalities e.g., neurological, cardiovascular, physical) on systolic and/or diastolic blood pressure, randomised control trial, published in English, adult participants aged 18 and over, with a diagnosis of hypertension (office reading of systolic blood pressure (SBP) ≥ 140 and/or diastolic blood pressure (DBP) ≥ 90 mmHg or home blood pressure readings of SBP ≥ 135 and/or DBP ≥ 85 mmHg) [14]. There was no specification for patients to be on specific types of hypertension treatment. Systematic reviews, editorial letters and conference abstracts were excluded.

### Study selection

The following electronic bibliographic databases were searched: PubMed, MEDLINE, PsycINFO, Embase, CINAHL and Cochrane Central Register of Trials. There was no date limit and all sources were last searched on January 16th, 2024. The search strategy followed the PICO criteria and was adjusted according to each database. The MEDLINE search strategy can be viewed in the Supplementary Material. Mendeley Desktop Reference Manager was used to store retrieved results and remove duplicates. Abstracts were reviewed in the first stage screening, which was completed by one review (S.J.), with a random 10% of abstracts screened by a second reviewer (A.C.). Disagreements were resolved after discussion between the two reviewers. The second stage screening reviewed the full text of articles and was completed by one reviewer (S.J.).

### Data extraction

Extracted data was retrieved and collated into an Excel spreadsheet by one reviewer (S.J.). Outcomes retrieved included participant characteristics, intervention design, study design, pre/post intervention measurements and conclusions. Please see the Supplementary Material for the list of outcomes and variables retrieved. The effect measure for all main outcomes was mean (±standard deviation).

### Synthesis methods

Studies were included in the meta-analysis if the mean and standard deviation was reported for a change in SBP and/or DBP. If reported, raw data and standard errors were converted to standard deviations and included. Authors were emailed for missing data and if there was no response, papers were excluded from the meta-analysis and assessed narratively.

The meta-analysis and forest plot diagrams were completed in Review Manager (version 5.4). A random effects model was used to assess systolic and diastolic blood pressure. Publication bias and Egger’s test was conducted in RStudio (version 2023.12.0 + 369).

A meta-regression was conducted on age and sex to explore possible causes of heterogeneity. However, there was insufficient data to conduct a reliable meta-regression for biofeedback modality. The meta-regression was a mixed effects model conducted in RStudio (version 2023.12.0 + 369).

### Data quality assessment

Papers were assessed for bias with the Cochrane Risk of Bias assessment [15], assessing for selection, reporting, performance, detection, attrition, and other sources of bias. The Risk of Bias 2 Tool [16] was used to input assessment, calculate summary data and figures, and to check inter-rater agreement.

The overall quality of evidence from reviewed studies was assessed with the GRADE assessment [17], which reviewed individual study limitations, inconsistency of results, indirectness of evidence, imprecision, and publication bias. The quality of evidence was rated from high to very low.

## Results

### Study selection

**Fig. 1:**
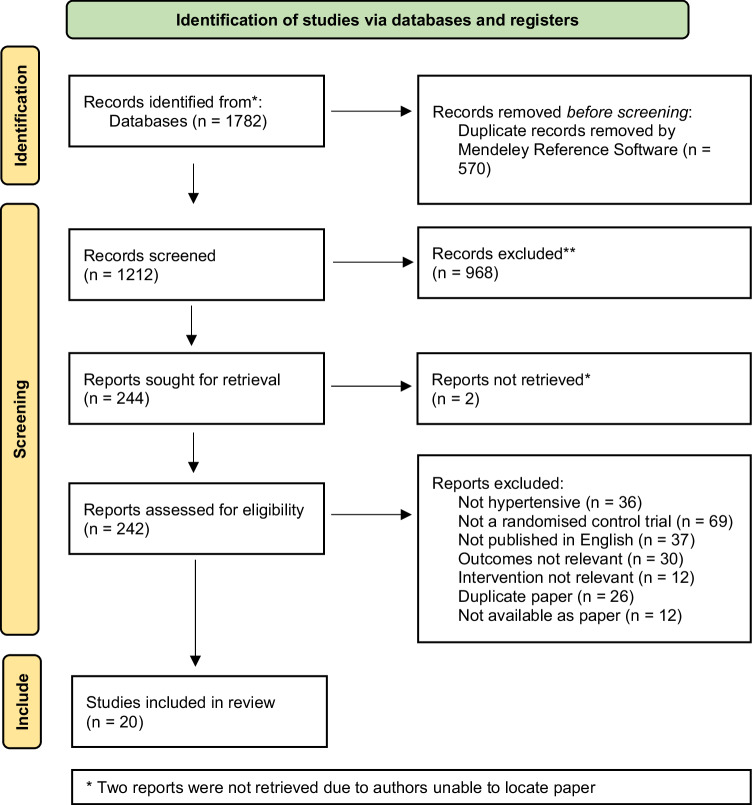
PRISMA flowchart. Flowchart in line with PRISMA guidelines indicating the number of articles originally identified, screened, excluded and included within the systematic review.

Figure 1 details the PRISMA flowchart. The search generated 1782 articles, with 244 potentially eligible articles identified during the title and abstract screening. The full text screen identified 20 articles that met the inclusion criteria for the review. Of these articles, 18 were from peer-reviewed journals and two were PhD theses. The main reasons for exclusion were study design not meeting the inclusion criteria (31%), articles not in English (18%), or outcomes outside of the inclusion criteria (14%).

### Participant characteristics

The overall characteristics of the 20 included studies are summarised in Table 1. The mean demographics are reported in Table 2. The studies were published between 1975 and 2013, from 10 different countries. There was a total of 988 participants and sample sizes ranged from 15 to 301 participants. The age ranged from 28 to 70 years, with 45% female participants. Ethnicity was reported in 3 articles, of these, 96% were Caucasian. The mean baseline SBP was 149.3 ± 7.8 mmHg and the mean DBP 93.0 ± 6.9 mmHg. Five studies [18–22] did not report data such as sex or age and were omitted from the above summary but were included in the main analysis as they reported key outcomes.

**Table 1:** Study characteristics and effect of biofeedback on blood pressure.

Author	Country	Sample (I/C)	Gender (% Female)	Age (Mean ± SD)	Baseline SBP (mmHg)	Baseline DBP (mmHg)	Change in SBP (mmHg)	Change in DBP (mmHg)
Fray [22]	USA	10/10	0	NR	N/A	I: 105.0 ± 9.4	N/A	I: 9.9
						C: 105.7 ± 9.0		C: 12
Patel et al. [33]	UK	17/17	61	I: 59.5^a^	I: 167.5 ± 23.6	I: 99.6 ± 9.3	I**: −26.1	I***: −15.2
				C: 58.6^a^	C: 168.9 ± 20.0	C: 100.6 ± 11.4	C: −8.9	C: −4.2
Blanchard et al. [20]	USA	19/9	NR	NR	I: 152.0	I: 103.4	I: −6.5	I: −2.6
					C: 145.4	C: 88.5	C: −9.7	C: −7.1
Billion [24]	USA	10/10	45	Total^b^: 52.4	I: 144.9 ± 16.8	I: 86.8 ± 8.2	I: −2.75	I: −5.3
					C: 142.6 ± 16.6	C: 87.3 ± 8.5	C: −2.25	C: −1.7
McGrady et al. [23]	USA	22/16	68	I: 55.0^a^	I: 144.4 ± 19.8	I: 90.5 ± 10.5	I**: −11.2	I**: −5.7
				C: 42.0^a^	C: 140.6 ± 19.4	C: 90.9 ± 11.7	C: −1.4	C: +0.8
Goldstein et al [26]	USA	9/9	17	I: 51.1^a^	I: 148.1^a^	I: 93.2^a^	I: −4.5	I: −3.9
				C: 49.1^a^	C: 136.5^a^	C: 89.9^a^	C: −0.8	C: +0.6
Hafner [19]	Australia	7/8	NR	Total^b^: 48.9	I: 160.0^a^	I: 106.6^a^	I: −20.8	I: −14.7
					C: 159.1^a^	C: 98.3^a^	C: −8.6	C: −2.0
Achmon et al. [35]	Israel	27/20	32	I: 40.1 ± 8.3	I: 155.0 ± 13.5	I: 99.7 ± 7.1	I**: −26.6	I**: −15.4
				C: 40.0 ± 8.6	C: 155.4 ± 20.0	C: 96.1 ± 6.3	C: −3.05	C: +0.8
Patel [25]	UK	49/54	49	NR	I: 144.9 ± 14.7	I: 88.6 ± 7.5	I**: −4.9	I: −1.5
					C: 135.7 ± 16.4	C: 85.1 ± 9.7	C: +7.1	C: +2.6
Canino et al. [38]	Venezuela	8/9	41	Total^b^: 35 ± 2	I: 147.0 ± 6.2	I: 96.0 ± 4.9	I: −13.0	I: −12.0
					C: 145.0 ± 8.9	C: 97.0 ± 6.5	C: 0	C: −1.0
Blanchard et al. [34]	USA	21/21	33	I: 50.0 ± 7.3	I: 142.1 ± 9.1	I: 93.2 ± 5.4	I: −1.2	I: −1.9
				C: 51.0 ± 6.8	C: 140.0 ± 14.6	C: 90.1 ± 6.0	C: +1.9	C: +1.0
Paran et al. [27]	Israel	20/12	44	I: 50.5 ± 8.7	I: 145.1^a^	I: 86.6^a^	I: −1.0	I: −0.8
				C: 46.8 ± 9.6	C: 147.2^a^	C: 94.6^a^	C: −1.6	C: −0.9
Hunyor et al. [18]	Australia	28/28	NR	18–69 (range)	I: 153.0 ± 9	N/A	I: −5.0	N/A
					C: 154.0 ± 8		C: −6.0	
Nakao et al. [32]	Japan	15/15	66	I: 55.0 ± 8	I: 158.0 ± 16	I: 95.0 ± 9	I**: −17	I**: −8.0
				C: 56.0 ± 8	C: 161.0 ± 21	C: 94.0 ± 6	C: +3.0	C: −1.0
Henderson et al. [28]	Australia	16/14	40	I: 55.0	I: 154.0 ± 10.6	I: 98.0 ± 4.3	I: −10.0	I: −7.0
				C: 53.0	C: 152.0 ± 6.5	C: 96.0 ± 3.3	C: −5.0	C: −3.0
Tsai et al. [29]	Taiwan	20/18	37	I: 46.5 ± 10.3	I: 148.4 ± 8.6	N/A	I***: −12.7	N/A
				C: 39.9 ± 10.8	C: 142.1 ± 5.9		C: −4.0	
Pandic et al. [30]	Sweden	31/22	73	I: 70.42 ± 8.7	I: 145.6 ± 15.0	I: 80.9 ± 10.6	I: −2.9	I: −1.5
				C: 66.5 ± 8.3	C: 151.8 ± 15.7	C: 82.7 ± 9.8	C: −16.8	C: −4.1
Olsson et al. [37]	Sweden	9/9	50	I: 52.5 ± 5.0	I: 154.6 ± 14.4	I: 96.6 ± 8.7	I: −5.9	I**: −7.6
				C: 60.6 ± 10.2	C: 148.7 ± 9.4	C: 92.0 ± 11.9	C: −0.8	C: −3.0
Landman et al. [31]	The Netherlands	21/24	38	I: 63.8 ± 7.9	I: 151.6 ± 8.3	I: 82.1 ± 4.7	I: −6.0	I: −5.9
				C: 65.1 ± 8.4	C: 151.2 ± 10.6	C: 80.7 ± 8.9	C: −8.4	C: −3.7
Elavally et al. [36]	India	150/151	44	NR	I: 140.6 ± 8.2	I: 88.2 ± 5.9	I***: −3.8	I***: −2.4
					C: 140.7 ± 10.2	C: 88.0 ± 5.1	C: +0.5	C: +0.7

This table has been arranged in date order.

*SBP* systolic blood pressure, *DBP* diastolic blood pressure, *N/A* not applicable.

**P < 0.05; ***P < 0.001.

^a^*SD* not available, *I/C* intervention/control.

^b^Total reflects overall age when age is not available for intervention and control group individually.

**Table 2:** Mean baseline measurements and demographics.

Measurement	Mean (± SD)
Age (year)	51.7 (±8.7)
Gender (Female)	45%
Baseline systolic blood pressure (mmHg)	149.3 (±7.8)
Baseline diastolic blood pressure (mmHg)	93.0 (±6.9)

### Types of biofeedback modalities

There were six different biofeedback modalities used across the 20 studies (Table 3). The type of biofeedback device used varied across studies and modalities, including finger or forehead electrodes [22–25], sphygmomanometer [18, 26, 27], finger blood pressure machines [28, 29] and compact disk (CD) players [30, 31]. No studies used a wearable device.

**Table 3:** Summary of biofeedback intervention components and design used in the included studies.

Study	Biofeedback modality	Follow up (weeks)	Number of sessions	Session length (mins)	Session type	Control group
Fray [22]	EMG	16	NR	NR	NR	Self-recorded measurements
Patel [33]	EMG and GSR	12	12	30	1 to 1	Placebo
Blanchard et al. [20]	EMG and BP	16	12	NR	1 to 1	Active control (self-instructed relaxation)
Billion [24]	EMG	NR	16	30	1 to 1	Placebo
McGrady et al. [23]	EMG	NR	16	30	1 to 1	Self-recorded measurements
Goldstein et al [26]	BP	NR	16	20	1 to 1	Self-recorded measurements
Hafner [19]	EMG and GSR	12	8	60	1 to 1	Self-recorded measurements
Achmon et al. [35]	HR	24	17	60	Groups of 4–13	Educational
Patel [25]	GSR	52	8	60	Groups of 10	NR
Canino et al. [38]	Thermal	24	15	75	1 to 1	Waiting list
Blanchard et al. [34]	Thermal	NR	16	30	Groups of 3–5	Self-recorded measurements
Paran et al. [27]	GSR and thermal	24	10	50	1 to 1	No biofeedback
Hunyor et al. [18]	BP	NR	8	12	1 to 1	Placebo
Nakao et al. [32]	BP	2	4	40	1 to 1	Self-recorded measurements
Henderson et al. [28]	SBP	NR	20	NR	Groups of 5–8	Placebo
Tsai et al. [29]	BP	8	4	45	1 to 1	No biofeedback
Pandic et al. [30]	RESPeRATE	NR	48	15	1 to 1	Active control (autogenic relaxation)
Olsson et al. [37]	Thermal	NR	8	12	1 to 1	Waiting list
Landman et al. [31]	RESPeRATE	NR	15	15	1 to 1	Placebo
Elavally et al. [36]	GSR	NR	4	30	1 to 1	Passive control (treatment as usual)

This table has been arranged in date order.

*SBP* systolic blood pressure, *BP* blood pressure, *EMG* electromyography, *GSR* galvanic skin response, *HR* heart rate, *RESPeRATE* branded version of auditory biofeedback, *NR* not reported.

Blood pressure biofeedback was used by six studies and was measured with either a non-invasive beat to beat finger arterial pressure measurement [29] or an automated blood pressure device [18, 20, 26, 27, 32]. Blood pressure biofeedback was typically received by the participant visually (e.g., a screen) [18, 26, 28, 29, 32] and/or auditorily (e.g., a beep) [22, 23]. For example, participants in the study by Tsai et al. [29] performed self-regulation techniques, such as deep breathing, and observed their blood pressure on a display.

Electromyographic (EMG) biofeedback detects changes or contractions in muscle. All six studies using EMG biofeedback gave auditory feedback, with some using the tone pitch and frequency to indicate EMG changes [19, 20, 22, 24, 33].

Galvanic skin response (GSR) biofeedback focuses on sweat gland activity and was used by five studies. As an example: Patel et al. [25] delivered the GSR feedback tone in one headphone and played a relaxation tape through the other headphone. The tone grew fainter as the participant relaxed and GSR activity reduced.

Thermal biofeedback was used in four studies. The intervention by Blanchard et al. [34] aimed to teach participants to increase temperature of their hands or feet, therefore strengthening deep-muscle relaxation.

RESPeRATE, a branded auditory based biofeedback device, was used in two studies [30, 31]. It involves listening and breathing in time with a melody to guide slow breathing [30].

Achmon et al. [35] was the only study to use heart rate biofeedback. It used ear lobe capillary pulsations to guide heart rate reductions in normal and tension-provoking situations.

### Intervention characteristics

Table 3 details the biofeedback intervention characteristics of the included studies. Biofeedback was mostly delivered one-to-one, with four studies delivering biofeedback to groups of 3–13 participants [19, 28, 33, 34]. Studies varied in the biofeedback session length (12–75 min) and number of sessions (4–48 sessions). The post-study follow up ranged from 2 weeks to 12 months, with ten studies not reporting any follow up. Biofeedback training was delivered by psychologists in four studies [27, 34, 36, 37] and by a trained nurse or therapist in two studies [32, 35]. Three studies used an experimenter or research assistant to deliver biofeedback [18, 20, 24], with the remaining eleven not detailing who delivered biofeedback training.

There were eight different control conditions used across studies, the most common were self-recorded blood pressure measurements and placebo. Six studies [22, 24, 26, 34, 35, 38] had multiple comparison groups (i.e., biofeedback and treatment as usual, placebo biofeedback, normotensive comparators). For data extraction, the treatment as usual group was prioritised as a comparator, followed by placebo biofeedback.

There was large variation across intervention design making it difficult to compare different designs and understand the most effective biofeedback intervention.

In terms of measuring how intervention delivery corresponded to the protocol, only two studies [29, 34] detailed methods that suggested fidelity checks, including a therapist remaining with the group throughout the intervention and a trained nurse implementing biofeedback under supervision of a qualified biofeedback practitioner. Only five studies [18, 25, 28, 31, 36] reported the use of power calculations to inform the sample size.

The methods used for blood pressure measurements varied across studies; seventeen [18–20, 22–25, 27–31, 33, 35–38] used clinic readings, and three [26, 32, 34] used home measurements. In the ten studies [19, 20, 23, 24, 27, 30–32, 35, 36] reporting medication use, 55% of participants were on anti-hypertensive medications. Medication status was not reported in three studies [25, 33, 37], whilst seven [18, 22, 26, 28, 29, 34, 38] studies reported participants were not taking any medications.

A total of seventeen studies detailed information regarding participant withdrawal or exclusion, with the remaining three studies [22, 26, 38] not reporting if any participants withdrew from the study. Overall, 111 participants withdrew, 44 were excluded and 2 participants died during the study time period. Reasons or details of participant withdrawal was limited, with 4 studies [19, 27, 33, 36] detailing if participants withdrew from the control or biofeedback group, and six studies detailing the specific stage participants withdrew at i.e., before or after baseline measurements [27, 37], after randomisation [30, 33, 35] or “within 2 weeks” [32]. Nine studies [18–20, 23, 24, 28, 29, 34, 36] did not detail at what stage participants withdrew. Reasons for participant exclusion included overly high blood pressure [28, 34], medication changes [23, 36], hypertrophy [18], failure in randomisation [25] and Olsson et al. [37] reported issues with biofeedback installation, commuting for the study and blood pressure not meeting hypertension criteria.

### Meta-analysis of suitable studies

A meta-analysis was conducted for SBP with twelve studies and DBP with eleven studies, since the remainder did not have adequate data as detailed in the methods section. The studies included in the meta-analysis had six different control conditions (please see Table 3).

**Fig. 2:**
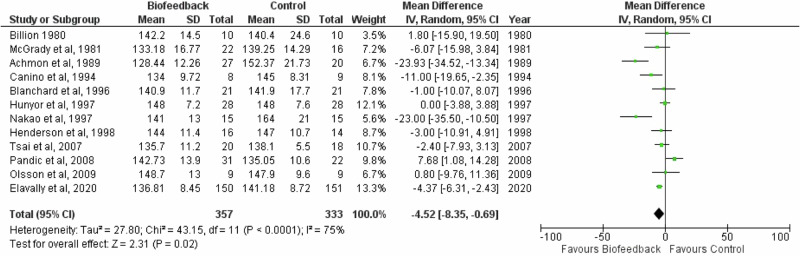
Forest plot of the effect of biofeedback on systolic blood pressure. The forest plot demonstrates a significant effect of biofeedback on systolic blood pressure.

**Fig. 3:**
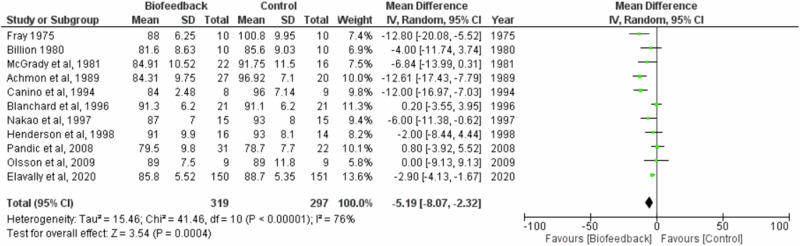
Forest plot of the effect of biofeedback on diastolic blood pressure. The forest plot demonstrates a significant effect of biofeedback on diastolic blood pressure.

The meta-analysis showed that biofeedback had a significant effect on SBP −4.52 (Z = 2.31, *P* = 0.02, CI [−8.35, −0.69]) and a significant effect on DBP −5.19 (Z = 3.54, *P* = 0.0004, CI [−8.07, −2.32] (Fig. 2). The forest plot shows heterogeneity was high for SBP I^2^ = 75% (Tau^2^ = 27.80; Chi^2^ = 43.15; *P* < 0.0001). The DBP forest plot can be seen in Fig. 3, also highlighting the high heterogeneity I^2^ = 76% (Tau^2^ = 15.46; Chi^2^ = 41.46; *P* < 0.00001).

Notably, Nakao et al. [32] and Achmon et al. [35] had substantial mean differences between the biofeedback and control group, with a mean difference in SBP of −23.00 mmHg in Nakao et al. [32] and −23.93 mmHg in Achmon et al. [35] studies. Within the papers there were limited reasons for the large decreases. Despite the large mean difference, neither paper was given a heavier weighting within the forest plot compared to other studies, with Nakao et al. [32] allocated 5.6% and 9.4% and Achmon et al. [35] allocated 6.7% and 10.0% for systolic and diastolic blood pressure respectively.

Additionally, it is noticeable that Pandic et al. [30] had a SBP mean difference of 7.68 mmHg in favour of the control group. The control group had a larger reduction in blood pressure compared to the RESPeRATE intervention group. The authors reflected on previous literature that showed relaxing music played to the control group can decrease blood pressure. Publication bias was assessed with Egger’s test and was non-significant for both SBP (−0.34, 95% CI [−2.22–1.54], *P* = 0.73) and DBP (−1.1, 95% CI [−3–0.75], *P* = 0.27). Corresponding funnel plots can be found in the Supplementary Material (Supplementary Figs. S1 and S2).

Of the eight studies excluded from the meta-analysis, only two showed significant findings in favour of biofeedback [19, 33] (Supplementary Tables S1 and S2). Across the 20 included studies, the pooled blood pressure difference in biofeedback groups was −9.5 mmHg SBP and −6.7 mmHg DBP, compared to −3.4 mmHg SBP and −1.9 mmHg DBP in control groups (Supplementary Table S3).

### Meta-regression

A meta-regression was conducted for age and sex on systolic and diastolic blood pressure. There was no significant association between participant age and effect of biofeedback on systolic (β = 0.49, SE = 0.40, 95% CI [−0.29, 1.26], *p* < 0.22) or diastolic (β = 0.44, SE = 0.26, 95% CI [−0.07, 0.96], *p* < 0.09) blood pressure (Supplementary Figs. S3 and S4).

There was no significant effect of sex on biofeedback outcomes, with no effect of participants being male on systolic (β = 0.00, SE = 0.05, 95% CI [-0.10, 0.11], *p* < 0.97) or diastolic (β = 0.01, SE = 0.04, 95% CI [−0.06, 0.07], *p* < 0.87) blood pressure, or being female on systolic (β = 0.01, SE = 0.07, 95% CI [−0.12, 0.15], *p* < 0.87) or diastolic (β = 0.01, SE = 0.05, 95% CI [−0.08, 0.10], *p* < 0.80) blood pressure (Supplementary Figs. S5–8).

### Quality assessments

The Cochrane risk of bias assessment identified there were “some concerns” (Supplementary Table S4). This was affected by 65% of papers not specifying randomisation allocation sequences or blinding of researchers or participants. All papers were raised to “some concerns” due to lack of pre-specified analysis plans.

The GRADE assessment showed data to have a “low certainty” of evidence, meaning further research is likely to change the estimate and have an important impact on confidence in the effect estimate. The certainty was downgraded from “high” to “low” due to inconsistency in evidence identified by heterogeneity and the risk of bias score. See Supplementary Material (Supplementary Table S5) for assessment ratings.

## Discussion

This was the first systematic review since 2009 to assess the effect of biofeedback in patients with hypertension (≥140/90 mmHg). The review and meta-analysis demonstrated that biofeedback significantly improved SBP and DBP. However, these results should be interpreted with caution given the limitations of included studies, such as heterogeneity, low study quality and limited details regarding randomisation, blinding and intervention delivery. The meta-regression analyses demonstrated that participant age or sex did not account for the heterogeneity seen within the meta-analysis.

The heterogeneity across biofeedback rendered it difficult to conduct modality specific analysis. Follow up ranged from 2 weeks to 12 months, with 10 studies not reporting if a follow up was conducted. Given the requirement of continued practice to benefit from biofeedback it is important to understand the longevity of the intervention [8].

Studies included in this review reporting using different instructors to deliver biofeedback to participants, including a psychologist, a trained therapist or nurse, a research assistant or experimenter. Eleven studies did not detail who delivered biofeedback. Although this review was unable to statistically compare delivery personnel and biofeedback outcomes, both studies which used nurse delivered biofeedback demonstrated a significant effect on blood pressure [32, 35]. For biofeedback to be a feasible and affordable intervention, the method and personnel delivering the intervention need to be considered. For services such as the NHS in the United Kingdom, it may benefit from biofeedback that is formulated to be delivered by a healthcare assistant or another allied health professional as this would be cheaper and scalable. More innovative solutions for delivery of biofeedback such as the use apps or videos need to be considered.

The results of this review are partly supported by the meta-analysis from Vital et al. [39] who included nine studies and found a significant reduction in DBP only. The current review differed from Vital et al. [39] as they included pre-hypertensive patients (SBP measuring 130–139 mmHg). The current review only included patients with SBP ≥ 140/90 mmHg because inclusion of patients with low-mild hypertension can lead to floor effects, with only small reductions possible [40]. An earlier review by Greenhalgh et al. [40] found no consistent evidence that demonstrated the benefits of biofeedback. However, they included thirty-six studies, some of which were excluded from the current systematic review based on low blood pressure readings (<140/90 mmHg), and missing or unclear outcomes. The inclusion of more studies, some of which did not meet the criteria for this review, may explain the higher heterogeneity and lack of consistent evidence in comparison to the present review.

This review is limited by heterogeneity and the number of studies included. This made it difficult to identify the most effective intervention design including, number of sessions, intervention length, and biofeedback modality. Despite the meta-analysis demonstrating a significant effect of biofeedback on SBP and DBP, the quality of data was low, especially relating to limited details on randomisation, blinding, missing pre-analysis statistical plans, and whether patients were on antihypertensive medications. The missing details regarding randomisation, blinding and key demographic data is a limitation that if submitted for publication in the current day, papers would not meet research guidelines. Limited details reported about interventions meant it was difficult to understand why some interventions worked, whilst others did not. Additionally, the lack of details regarding at what stage participants withdrew from the study make it difficult to understand if withdrawal was due to the requirements of biofeedback, or for another reason. Similarly, the wide variation in control conditions add difficulty to understanding the effect of biofeedback. This poor quality of data is similar to the findings of by previous reviews, highlighting the need for improved methods and reporting in future studies.

The included studies have several limitations that may affect the reliability of outcomes. These include wide variations in sample size, which may result in findings that do not reflect real patients. In line with representation, the mean age of the included participants was 51.7 ± 8.7 years, which does not reflect the mean age of patients with hypertension, which largely affects patients aged over 65 [41]. Only 25% of articles reported any power calculations. No studies reported measurements of medication adherence, which can significantly affect blood pressure control [42, 43]. Furthermore, ten studies used participant populations that were either partly, or not on any medication. Similarly, intervention adherence was reported in only four studies, and two studies reported the use of fidelity checks. Consequently, it is difficult to ensure the biofeedback intervention was implemented as planned in the majority of studies.

A significant issue in this review is that the dated studies do not represent the availability of current technologies. The majority of studies were published between 1970 and 1999, with only one study published after 2010. Since then, biofeedback technology has improved dramatically and is more user friendly, with the ability to practice independently at home. This has been further supported by the progression with AI, which can further support the development and integration of biofeedback in the healthcare field. It has already been incorporated in biofeedback research in virtual reality exposure therapy for anxiety [44] and eXtended Reality training scenarios [45]. The use of machine learning in biofeedback can support tailored feedback and identify scenarios and stimuli that increase physiological responses, which can increase user awareness of their health. Biofeedback devices now include wearables, such as a wristband that continuously records blood pressure and displays results in an app on the user’s phone [11]. This contrasts with examples from included studies where biofeedback was conducted in the clinic in the presence of researchers [29, 34] and using techniques not suitable for home use, such as a researcher manually plotting blood pressure biofeedback on a graph [20]. The dated technology is reflected in methods of blood pressure measurement.

We believe that biofeedback has a potential role to play in the management of hypertension. New research should incorporate robust methodology, updated biofeedback technology such as wearable devices, and incorporate the use of innovative techniques to support large scale delivery of biofeedback.

To conclude, this meta-analysis showed that biofeedback significantly reduces systolic (-4.52 mmHg, *P* = 0.02) and diastolic blood pressure (−5.19 mmHg, *P* = 0.0004), with the pooled blood pressure decrease in biofeedback groups reaching clinical significance. However, the low quality of evidence and heterogeneity across studies mean results should be interpreted with caution. Importantly, the dated nature of existing studies means they do not represent the current climate of biofeedback and the availability of current technologies. But future research especially featuring wearable devices using robust methodology are needed to provide evidence of a practical and scalable approach to biofeedback that is clinically deliverable and acceptable to patients.

## Summary

### What is known about the topic

- Hypertension is a leading modifiable risk factor for cardiovascular disease. However, despite the availability of medication, hypertension control remains suboptimal in approximately 50% of patients.
- Autonomic nervous system dysregulation in part mediates hypertension, highlighting a possible target for interventions aiming to improve blood pressure.
- Biofeedback can increase self-regulation and self-awareness of parameters that regulate the autonomic nervous system, suggesting a suitable intervention to support patients with hypertension

### What this study adds

- The meta-analysis demonstrated that biofeedback had a significant effect on blood pressure, with a reduction in both systolic (−4.52, Z = 2.31, *P* = 0.02, CI [−8.35, −0.69]) and diastolic blood pressure (−5.19, Z = 3.54, *P* = 0.0004, CI [−8.07, −2.32]).
- The weaknesses of the study not only make it difficult to determine the most effective intervention but also affect the ability to draw conclusions about the effect of biofeedback on blood pressure.
- Future studies need to incorporate robust methodology and updated technology such as wearable devices, to improve understanding of the role of biofeedback in hypertension.

## Supplementary information

Supplementary material

## Data Availability

All data generated or analysed during this review are included in this published article [and its supplementary information files].
