# Supplementary material for: Effectiveness of biofeedback on blood pressure in patients with hypertension: systematic review and meta-analysis
Source: J Hum Hypertens. 2024 Aug 14;38(10):719–27. doi: 10.1038/s41371-024-00937-y (PMC11458477; doi:10.1038/s41371-024-00937-y)

Supplementary material

Supplementary methods

MEDline search strategy

exp Biofeedback, Psychology/
biofeedback.mp.
BFB.mp.
1 or 2 or 3
high blood pressure.mp.
uncontrolled blood pressure.mp.
blood pressure.mp. or Blood Pressure/
diastolic blood pressure.mp.
systolic blood pressure.mp.
(BP or SBP or DBP or diastolic BP or systolic BP).mp. [mp=title, abstract, original title, name of substance word, subject heading word, floating sub-heading word, keyword heading word, organism supplementary concept word, protocol supplementary concept word, rare disease supplementary concept word, unique identifier, synonyms]
5 or 6 or 7 or 8 or 9 or 10
exp Emotions/ or Depression/ or "Quality of Life"/ or emotional wellbeing.mp. or Mental Health/
psychological wellbeing.mp.
depression.mp.
exp Anxiety/ or anxiety.mp. or exp Anxiety Disorders/
exp Stress, Psychological/ or exp Stress, Physiological/ or stress.mp. or exp Occupational Stress/
mental health.mp.
mood.mp. or exp Affect/
11 or 12 or 13 or 14 or 15 or 16 or 17
4 and 11 and 19
4 and (11 or 19)

List of variables and outcomes retrieved

- Paper details
    - Authors
    - Publication dates
    - Outcomes and measurements
    - Comparator group
    - Participant diagnosis
- Participant characteristics
    - Sample size
    - No. participants dropped out
    - Age (Mean ± SD)
    - Gender (M/F)
    - Ethnicity
    - BMI (Mean ± SD)
    - Participants on medication
    - Medication type
- Intervention details
    - Type of biofeedback
    - Biofeedback details
    - Validated biofeedback measurement
    - Length of intervention
    - Number of sessions
    - Follow up period
    - Randomisation
    - Power calculations
    - Calculations for confounding variables
- Outcome measurements
    - Systolic blood pressure (mmHg) (Mean ±SD) (pre intervention, post intervention, follow up)
    - Diastolic blood pressure (mmHg) (Mean ±SD) (pre intervention, post intervention, follow up)
- Conclusions
    - Proposed theory/mechanisms
    - Reasons for change/lack of change
    - Conclusions
    - Other
- Limitations

Tables

Table S1. Narrative summary of remaining systolic blood pressure studies

Author	Biofeedback modality	Biofeedback pre (mean±SD)	Biofeedback post (mean)	Control pre (mean±SD)	Control post (mean)	Significance
Patel et al (1975)	GSR and EMG	167.5±23.6	141.4	168.9±20.0	160	P<0.005
Blanchard et al (1979)	EMG and BP*	152.0	145.5	145.4	135.7	NS
Goldstein et al (1982)	BP	148.1	143.6	136.5	135.7	NS
Hafner et al (1982)	GSR and EMG	160	139.2	159.1	150.5	NS
Patel et al (1988)	GSR	144.9±14.6	140	135.7±16.44	142.8	P=0.007
Paran et al (1996)	GSR and thermal	145.1	146.1	147.2	145.6	NS
Landman et al (2013)	RESPeRATE	151.6±8.3	145.57	151.2±10.6	142.82	NS
Galvanic skin response (GSR); EMG (electromyography); BP (blood pressure); RESPeRATE (brand of auditory breathing-based biofeedback); NS (non-significant)						

Table S2. Narrative summary of remaining diastolic blood pressure studies

Author	Biofeedback modality	Biofeedback pre (mean±SD)	Biofeedback post (mean)	Control pre (mean±SD)	Control post (mean)	Significance
Patel et al (1975)	GSR and EMG	99.6±9.3	84.4	100.6±11.4	96.4	P<0.001
Blanchard et al (1979)	EMG and BP	103.4	100.8	88.5	81.4	NS
Goldstein et al (1982)	BP	93.2	89.3	89.9	90.5	NS
Hafner et al (1982)	GSR and EMG	106.6	91.9	98.3	96.3	NS
Patel et al (1988)	GSR	88.6±7.5	87.1	85.1±9.67	87.7	P=0.131
Paran et al (1996)	GSR and thermal	86.6	85.8	94.6	95.5	NS
Landman et al (2013)	RESPeRATE	82.1±4.7	76.18	80.7±8.9	77.03	NS
Galvanic skin response (GSR); EMG (electromyography); BP (blood pressure); RESPeRATE (brand of auditory breathing-based biofeedback); NS (non-significant)						

Table S3: Pooled mean differences of all included studies

	Biofeedback (Mean±SD)		Pooled difference	Control (Mean±SD)		Pooled difference
	Pre	Post		Pre	Post	
SBP (mmHg)	150.4±12.92	140.9±11.93	-9.5	148.3±13.55	144.9±13.36	-3.4
DBP (mmHg)	93.9±7.54	87.2±7.92	-6.7	92.1±8.15	90.1±8.19	-1.9

Table S4. Summary of the Cochrane risk of bias assessment for the included studies

Paper	Domain 1	Domain 2	Domain 3	Domain 4	Domain 5	Overall
Fray, 1975	!	+	+	+	!	!
Patel et al, 1975	!	+	+	+	!	!
Blanchard et al, 1979	!	+	+	+	!	!
Billion, 1980	!	+	+	+	!	!
McGrady et al, 1981	!	+	+	+	!	!
Goldstein et al, 1982	!	+	+	+	!	!
Hafner, 1982	!	+	+	+	!	!
Achmon et al, 1987	!	+	+	+	!	!
Patel et al, 1988	+	+	+	+	!	!
Canino et al, 1994	!	+	+	+	!	!
Blanchard et al, 1996	!	+	+	+	!	!
Paran et al, 1996	!	+	+	+	!	!
Hunyor et al, 1997	+	+	+	+	!	!
Nakao et al, 1997	!	+	+	+	!	!
Henderson et al, 1998	!	+	+	+	!	!
Tsai et al, 2007	+	+	+	+	!	!
Pandic et al, 2008	+	+	+	+	!	!
Olsson et al, 2009	+	+	+	+	!	!
Landman et al, 2013	+	+	+	+	!	!
Elavally et al, 2020	+	+	+	+	!	!
Key: Low risk (+); Some concerns (!); High risk (-)						

Table S5. Summary results of GRADE assessment showing the overall quality of included studies

Domain	Rating
1. Individual study limitations	Serious
2. Inconsistency of results	Serious
3. Indirectness of evidence	Not serious
4. Imprecision	Not serious
5. Publication bias	Not serious
Overall decision	Low certainty of evidence

Figures

Figure S1. Funnel plot to demonstrate publication bias in systolic blood pressure articles

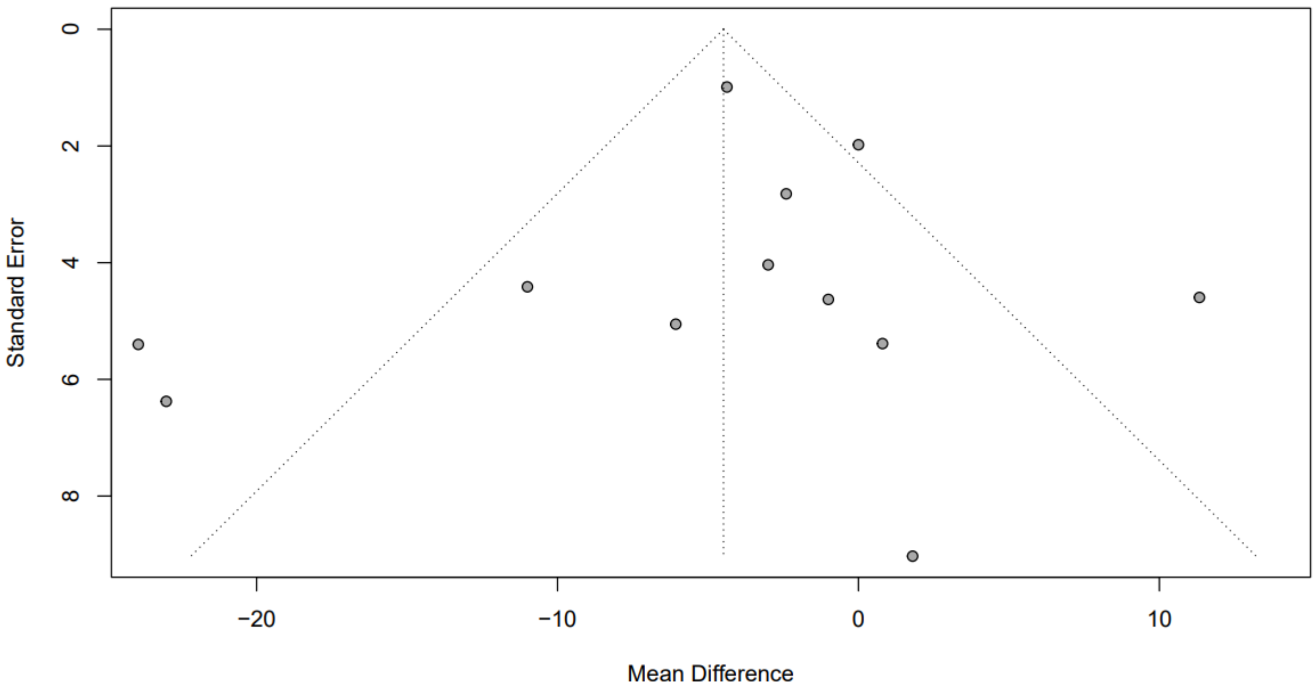

Figure S2. Funnel plot to demonstrate publication bias in diastolic blood pressure articles

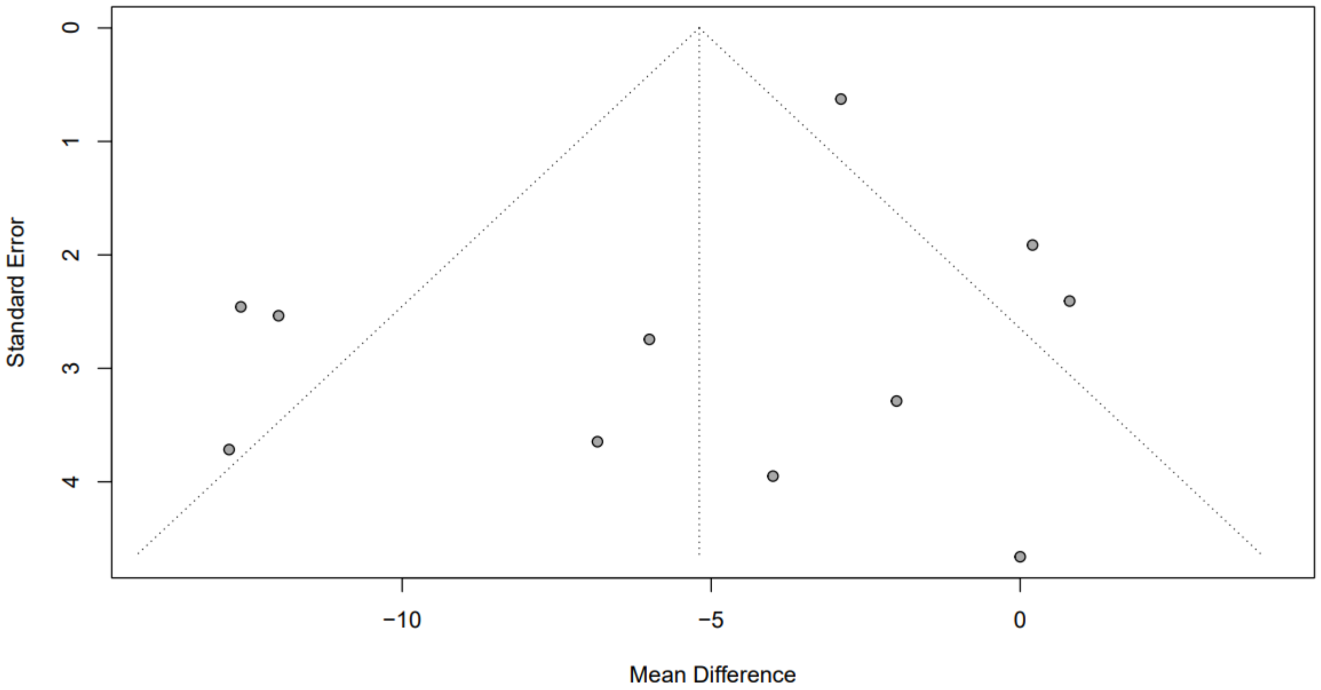

Figure S3: Bubble plot to demonstrate the meta-regression of systolic blood pressure and age

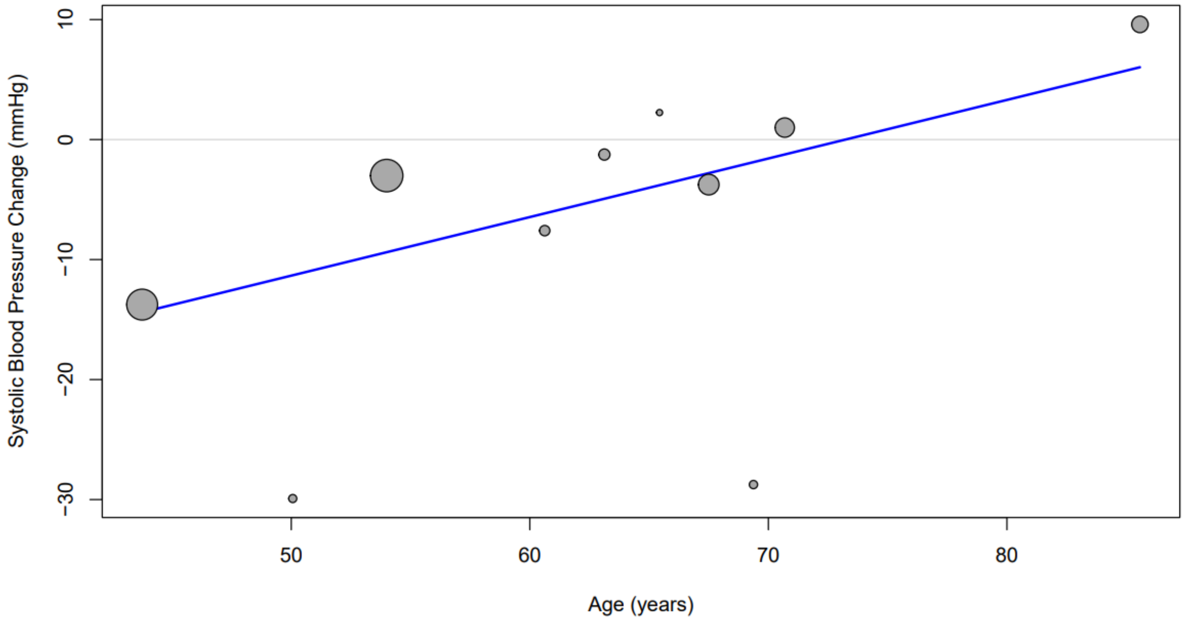

Figure S4: Bubble plot to demonstrate the meta-regression of diastolic blood pressure and age

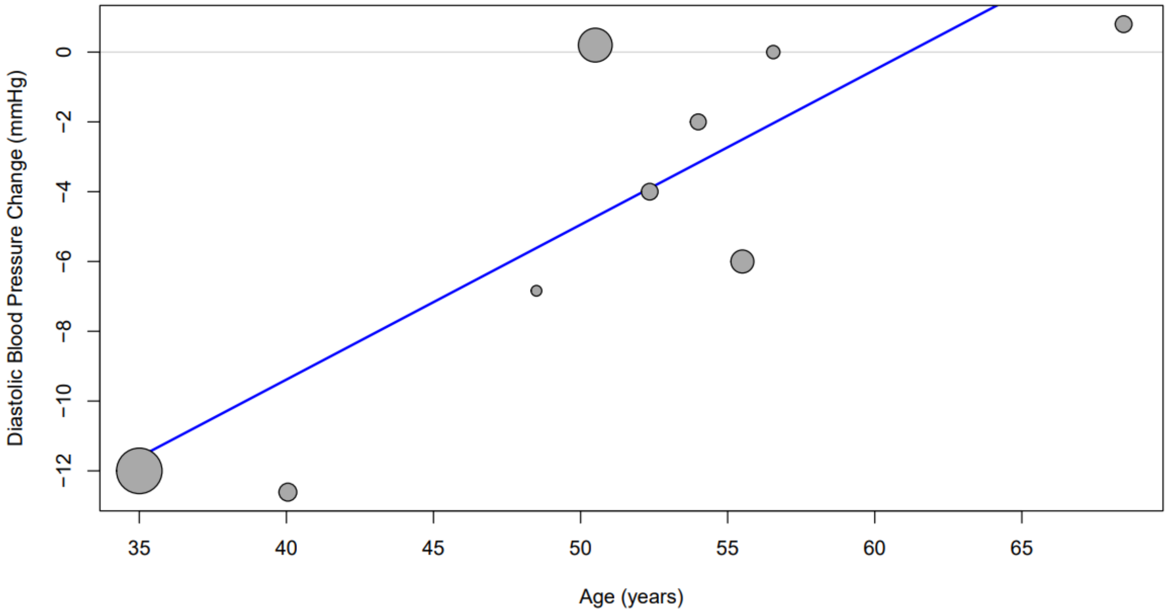

Figure S5: Bubble plot to demonstrate the meta-regression of systolic blood pressure and sex (male)

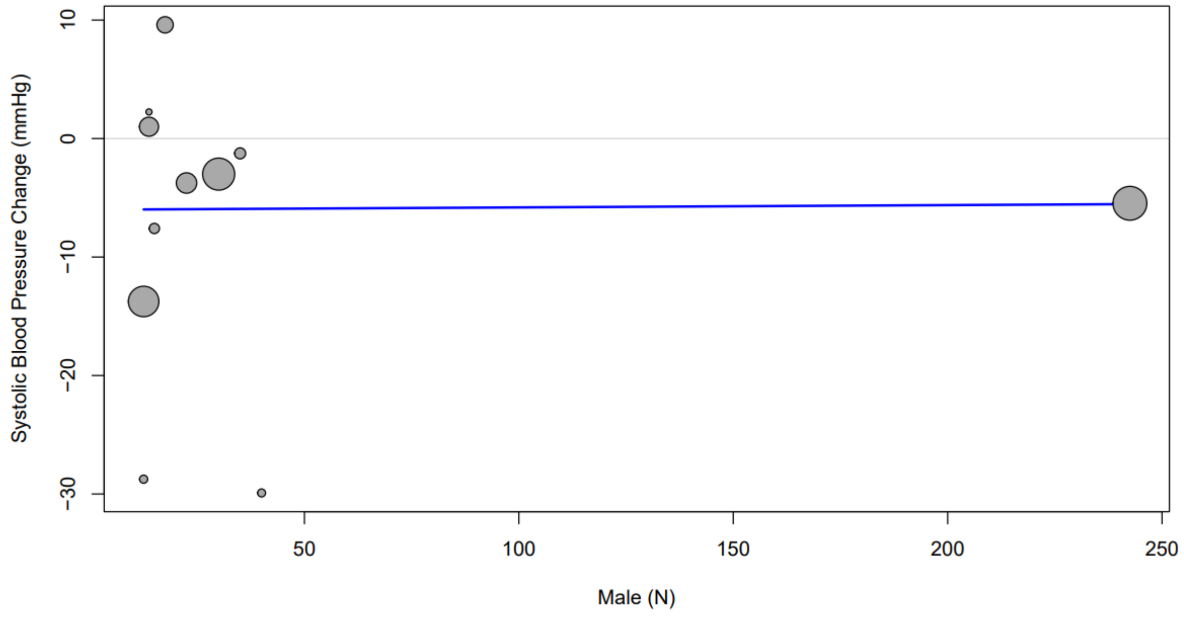

Figure S6: Bubble plot to demonstrate the meta-regression of diastolic blood pressure and sex (male)

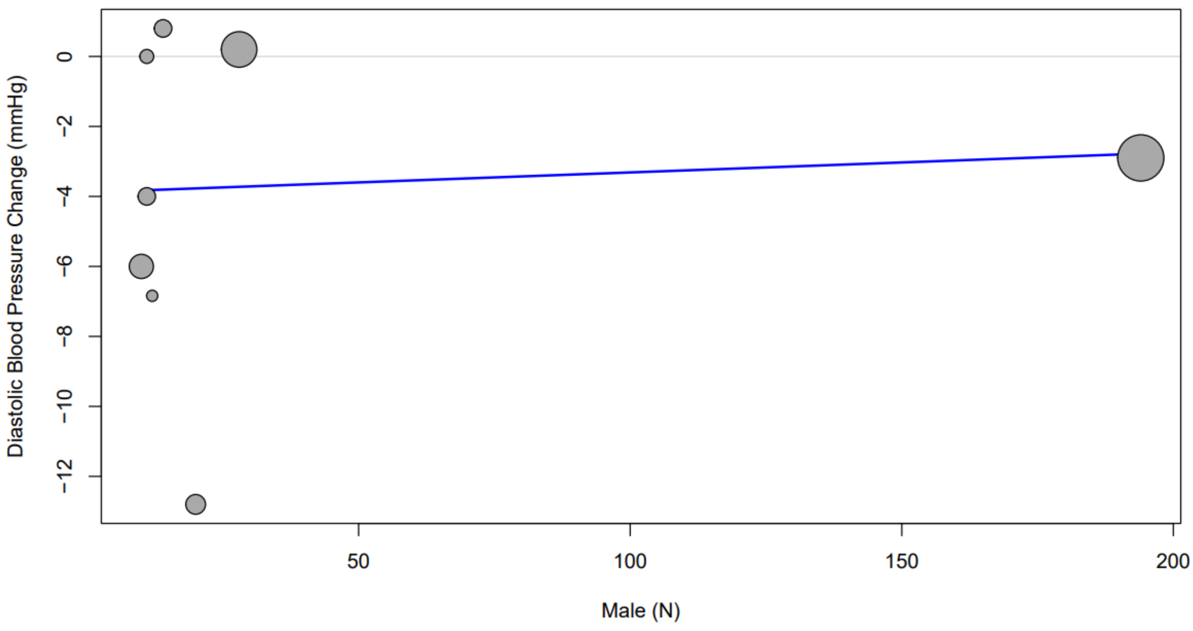

Figure S7: Bubble plot to demonstrate the meta-regression of systolic blood pressure and sex (female)

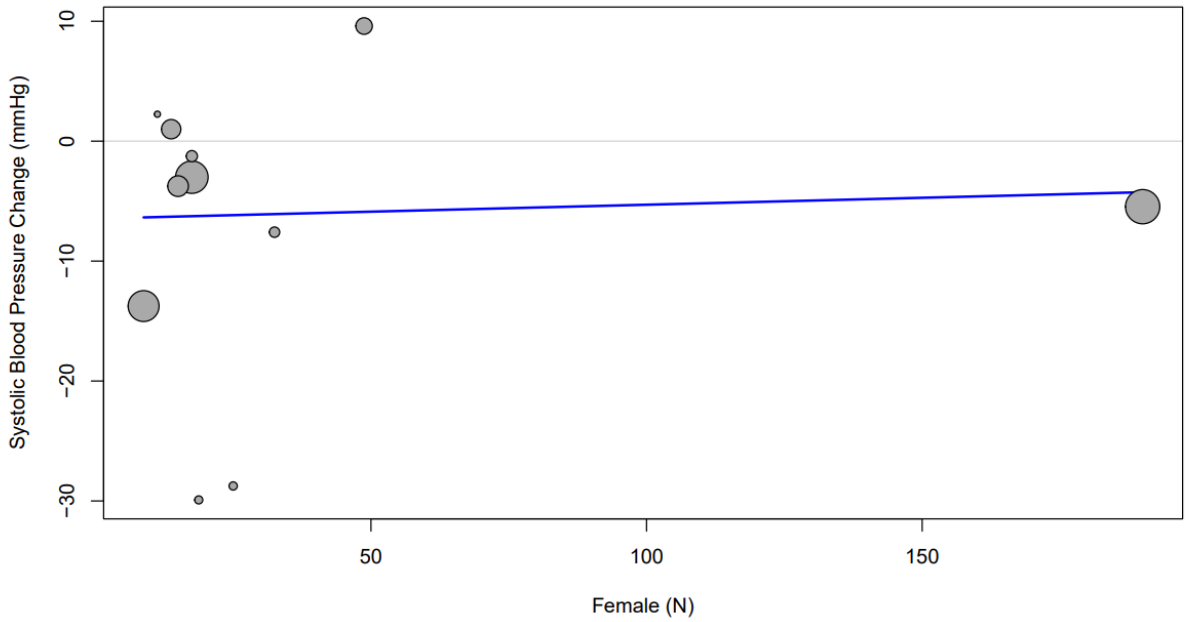

Figure S8: Bubble plot to demonstrate the meta-regression of diastolic blood pressure and sex (female)

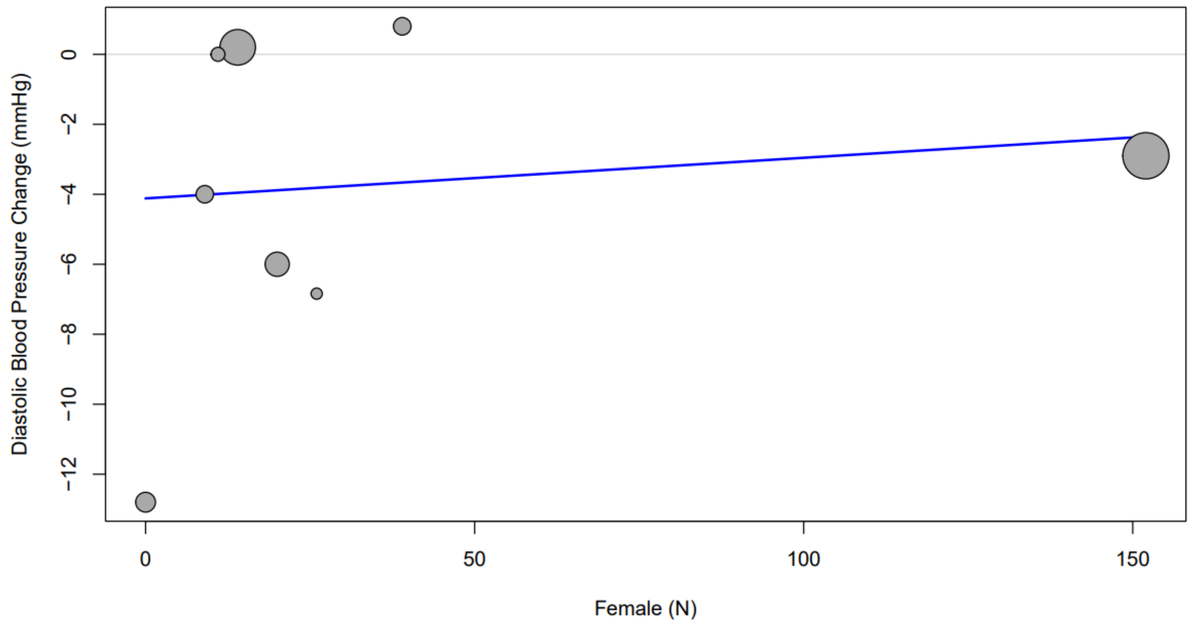

Supplement: Supplementary file 1 — Supplementary material [file 41371_2024_937_MOESM1_ESM.pdf]
